# Targeting the gastrin-releasing peptide receptor pathway to treat cognitive dysfunctionassociated with Alzheimer’s Disease

**DOI:** 10.1590/s1980-57642008dn10200002

**Published:** 2007

**Authors:** Rafael Roesler, Tatiana Luft, Gilberto Schwartsmann

**Affiliations:** 1 Cellular and Molecular Neuropharmacology Research Group, Department of Pharmacology, Institute for Basic Health Sciences, Federal University of Rio Grande do Sul, 90046-900 Porto Alegre, RS, Brazil.; 2 Cancer Research Laboratory, Academic Hospital Research Center, Federal University of Rio Grande do Sul, 90035-003 Porto Alegre, RS, Brazil.; 3 Department of Biochemistry, Institute for Basic Health Sciences, Federal University of Rio Grande do Sul, 90035-003 Porto Alegre, RS, Brazil.; 4 Department of Internal Medicine, Faculty of Medicine, Federal University of Rio Grande do Sul, 90035-003 Porto Alegre, RS, Brazil

**Keywords:** bombesin-like peptides, gastrin-releasing peptide, gastrin-releasing peptide receptor, cognitive enhancers, memory disorders, Alzheimer disease

## Abstract

Increasing evidence indicates that bombesin (BB)-like peptides (BLPs), such as the gastrin-releasing peptide (GRP) and its receptor (GRPR), might play a role in neurological and psychiatric disorders. The present study reviews findings from animal and human studies suggesting that the GRPR should be considered a target for the treatment of cognitive dysfunction in patients with Alzheimer’s disease (AD). Abnormalities in GRPR-triggered signaling have been described in both fibroblasts from patients with AD, and in transgenic mouse models of AD. Pharmacological and genetic preclinical studies have indicated that BLPs and the GRPR are importantly involved in regulating cognitive function. Moreover, drugs acting at the GRPR have been shown to enhance memory and ameliorate cognitive dysfunction in experimental models of amnesia associated with AD. Taken together, these findings support the view that the GRPR is a novel therapeutic target for the treatment of memory deficits associated with AD.

## Abstract (pt)

Estudos recentes indicam que os peptídeos da família da bombesina (BB), como o peptídeo liberador de gastrina (GRP) e seu receptor (GRPR), podem estar envolvidos em doenças neurológicas e psiquiátricas. Este artigo apresenta uma revisão de estudos tanto em humanos como em modelos animais que sugerem que o GRPR deve ser considerado um alvo molecular para o desenvolvimento de novas terapias para o tratamento de déficits cognitivos em pacientes com doença de Alzheimer (DA). Anormalidades na sinalização celular dependente do GRPR têm sido descritas tanto em fibroblastos de pacientes com DA como em modelos de DA em camundongos transgênicos. Além disso, estudos pré-clínicos utilizando estratégias farmacológicas e genéticas indicam que os peptídeos da família da BB e o GRPR estão envolvidos de forma importante na regulação da função cognitiva. Finalmente, resultados recentes mostram que drogas que agem como ligantes do GRPR podem melhorar a memória e prevenir disfunções cognitivas em modelos experimentais de amnésia asssociada à DA. Em conjunto, os dados indicam que o GRPR é um novo alvo terapêutico para o tratamento de déficits de memória associadas à DA.

## Bombesin-like peptides and their receptors in the brain

Bombesin (BB) is a 14 amino acid initially isolated from the skin of frogs *Bombina bombina*. It was later described that gastrin-releasing peptide (GRP), a 27 amino acid peptide functionally and structurally related to BB, is a mammalian counterpart of BB (Table 1). BB and GRP, as well as other related peptides such as neuromedin (NM) B (NMB), constitute a family of BB-like peptides (BLPs). BLPs have been described to affect a range of cellular and neuroendocrine functions, including cell proliferation and differentiation, cancer growth, feeding behavior, and stress responses (for recent reviews, see^1-4^) .

**Table 1:** Structures of bombesin (BB) and gastrin-releasing peptide (GRP).

**Bombesin**
Pyr-Gln-Arg-Leu-Gly-Asn-Gln-Trp-Ala-Val-Gly-His-Leu-Met-NH2
**Gastrin-releasing peptide**
Ala-Pro-Val-Ser-Val-Gly-Gly-Gly-Thr-Val-Leu-Ala-Lys-Met-Tyr-Pro-Arg-Gly-Asn-His-Trp-Ala-Val-Gly-His-Leu-Met-NH2

Adapted from ^[1,4,7]^.

Early studies investigating the presence of BB binding sites in the mammalian central nervous system (CNS) showed that BB bound with high affinity to rat brain membranes. The hippocampus, a brain area critically involved in cognitive function and neurodegenerative and neuropsychiatric disorders, including Alzheimer’s disease (AD), had the highest density of specific BB binding sites.^5^ Subsequent studies identified the occurrence of endogenous BLPs as neuropeptides in the rat CNS. It is now well established that GRP, the main mammalian BLP, is like a co-transmitter released from both central and peripheral neurons that regulates aspects of brain function including memory and emotional processing (for reviews, see^1,4^) (Table 1).

## The gastrin-releasing peptide (GRPR) receptor and associated signal transduction pathways

BB and GRP exert most of their biological actions by binding at the GRP receptor (GRPR, also known as BB2 receptor). GRPR is a member of the G-protein coupled receptor superfamily containing seven transmembrane domains and 384 amino acids.^6-8^ GRPR is highly expressed in the brain. Studies using in vitro autoradiographic techniques have indicated that brain areas containing high densities of GRPRs include the olfactory bulb, nucleus accumbens, caudate putamen, central amygdala, dorsal hippocampal formation, as well as the paraventricular, central medial, and paracentral thalamic nuclei.^1,4,9,10^ A recent seminal immunohistochemical study has used affinity-purified GRPR antibodies to examine the precise distribution of GRPR in the mouse brain. GRPR immunoreactivity was widely distributed in the isocortex, hippocampus, piriform cortex, amygdala, hypothalamus, and brain stem, with high concentrations in the dorsal hippocampus and lateral amygdala. In addition, GRPR expression was specific for the cell membranes of neuronal dendrites and cell bodies.^11^

Intracellular responses to GRPR activation were initially examined in cancer and neuroendocrine cell lines. Cellular signaling pathways for the GRPR have been shown to include protein kinase signaling cascades, particularly the protein kinase C (PKC) and mitogen-activated protein kinase (MAPK)/extracellular signal-regulated protein kinase (ERK) pathways.^12-14^ In the brain, GRP-induced neuronal membrane depolarization in the rat hippocampus is blocked by a PLC inhibitor,^15^ and we have recently shown that modulation of the rat hippocampal function by BB depends on the PKC, MAPK and PKA pathways (Figure).^16^

**Figure:**
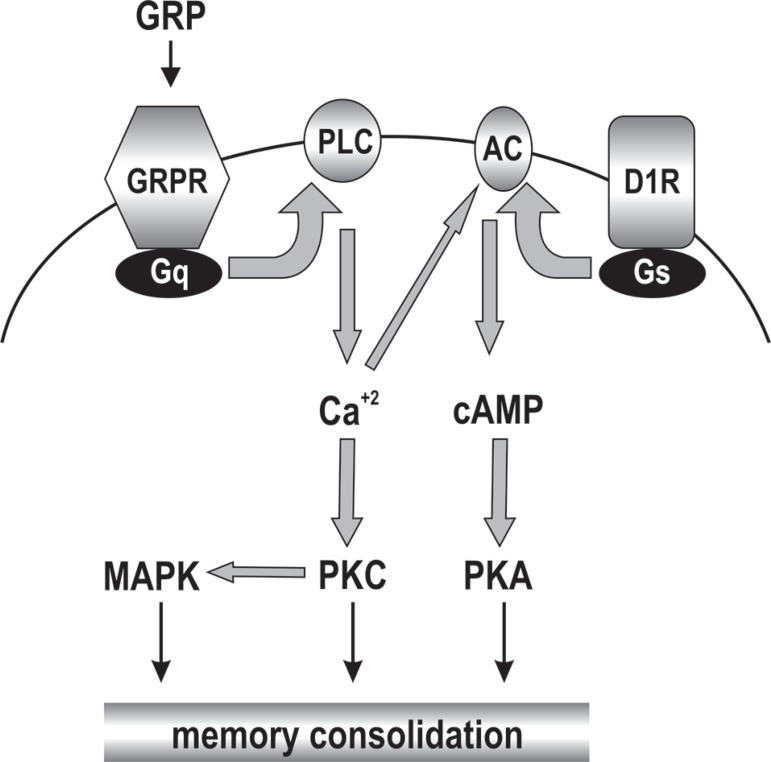
Proposed signaling pathways associated with the gastrinreleasing peptide receptor (GRPR) in the central nervous system. Gastrin-releasing peptide (GRP) released from synaptic terminals binds to the G_q_ protein-coupled GRPR at postsynaptic sites. GRPR activation induces an increase in [Ca^2+^] and triggers activation of the phospholipase C (PLC)/protein kinase C (PKC) pathway, which, in turn, can activate mitogen-activated protein kinase (MAPK). The dopamine D1/D5 receptor (D1R) is coupled to Gs protein (G_s_) and adenylyl cyclase (AC) activation. The D1R-induced cAMP signal might be synergistically potentiated by [Ca^2+^]-induced stimulation of [Ca^2+^]-responsive types of AC, leading to increased activation of protein kinase A (PKA). Reproduced from [16], with permission.

An increasing body of evidence indicates that BLPs and the GRPR might play a role in CNS disease, including memory disorders associated with AD and other neurodegenerative disorders. Thus, our group has put forward the GRPR as a novel therapeutic target for the development of therapies to treat neurological and psychiatric disorders.^4,17^ The present study reviews current evidence suggesting the GRPR should be considered a target for the treatment of cognitive dysfunction in patients with AD.

## Abnormalities in GRPR function in Alzheimer’s disease: evidence from mice and human studies

Increasing evidence from animal and human studies has indicated that abnormalities in BLPs- and GRPR-triggered cellular signaling might be associated with AD. Dysregulation of calcium signaling has been causally implicated in both normal brain aging and AD. BB stimulates calcium release from BB-releasable calcium stores in the endoplasmic reticulum (ER). Exaggerated BB-induced intracellular calcium release has been demonstrated in fibroblasts and neurons from genetically modified mice bearing a mutation in the presenilin-1 (PS-1) mutation.^18^ These transgenic mice have been developed as a useful animal model since mutations in the presenilin-1 (PS1) gene on chromosome 14 are causally linked to many cases of early-onset inherited AD.^18,19^ Importantly, the alterations in BB-induced enhancement of calcium signaling observed in this mouse model resemble those described in patients with AD. Both increased and reduced calcium signals have been described in AD patients. Thus, fibroblasts from familial and non-familial AD cases have shown enhanced calcium signals induced by BB compared to controls.^20-24^ In contrast, in fibroblasts from patients with familial Alzheimer’s disease presenting the Swedish APP670/671 mutation, BB-induced elevations in calcium were found to be reduced by 40%.^21^ These abnormalities in BB-regulated calcium homeostasis observed in AD fibroblasts have been proposed to involve alterations in oxidative stress.^20-23,25^ Since alterations in calcium signaling and oxidative stress might be involved in neurodegeneration and cognitive impairment in AD patients, these findings from mouse and human studies support the view that BLP-triggered signaling and the GRPR pathway might play a role in the pathogenesis of AD.

Another cellular change related to BLP- and GRPR-elicited signaling described in fibroblasts from patients with AD, is a reduced number of BB receptors.^24^ This interesting finding raises the possibility that decreased neuronal GRPR density, leading to impaired BLP function in the brain of AD patients, is related to neurodegeneration and memory loss associated with the disease. Table 2 summarizes relevant alterations in the GRPR pathway observed in patients with AD (Table 2).

**Table 2:** Abnormalities in the gastrin-releasing peptide receptor (GRPR) pathway in patients with Alzheimer's disease (AD).

Finding	References
Enhanced bombesin (BB)-induced calcium release in fi brobasts	^[23, 24]^
Reduced BB-induced calcium mobilization in fibrobasts in patients with the Swedish APP670/671 mutation	^[21]^
Increased response of BB-induced calcium release to oxidant agents in patients with presenilin-1 (PS-1) mutation	^[23]^
Reduced number of gastrin-releasing peptide receptors (GRPRs) in fi broblasts	^[24]^

## Effects of drugs acting at the GRPR on cognitive function: preclinical findings

The present and other authors have used rodent models of learning and memory to investigate the role of brain BLPs and the effects of drugs acting at the GRPR in cognitive function. Systemic administration of BB or GRP enhances memory retention in rats and mice,^26,27^ whereas injections of GRPR antagonists cause impairment.^28-32^ GRPR agonists and antagonists also modulate memory formation and extinction when infused intracranially into specific brain areas.^16,31,33-38^ For instance, GRPR inactivation in either the dorsal hippocampus or basolateral amygdala by infusions of the selective GRPR antagonist [D-Tpi,^6^ Leu^13^ psi(CH_2_NH)-Leu^14^] bombesin (6-14) (RC-3095), a synthetic BB analog, hinders retention of memory for inhibitory avoidance, a type of fear conditioning-based task, in rats.^31,36,37^ Moreover, the findings from pharmacological studies are supported by genetic evidence showing altered memory formation and synaptic plasticity in GRPR-deficient knockout mice.^39^

Our group has shown that the dorsal hippocampus is a brain area crucially involved in mediating the regulatory actions of BLPs on memory.^16,33,34,36,37^ Importantly, microinfusion of BB into the rat CA1 hippocampal area has enhanced inhibitory avoidance consolidation. We went on to investigate the molecular mechanisms mediating the memory-enhancing effect of intrahippocampal BB administration. BB-induced modulation of memory consolidation was prevented by infusion of a GRPR antagonist or inhibitors of the PKC, MAPK, and PKA signaling pathways. These findings indicated that BB (and presumably other BLPs) might facilitate cognitive function by activating GRPRs in hippocampal neuronal membranes, thus leading to activation of intracellular signal transduction pathways known to mediate synaptic plasticity and memory formation.^16^ Other experiments have suggested that the GRPR signaling system might have functional interactions with glucocorticoid receptors^37^ and inhibitory neurons releasing gamma-aminobutyric acid (GABA)^33^ in regulating memory formation in the hippocampus.

## Prevention of memory impairment induced by the Alzheimer peptide through a GRPR agonist in a rat model

Our findings described above, that BB can stimulate cellular signaling mechanisms that mediate synaptic plasticity and enhance memory formation, suggest that BLPs should be further evaluated as potential cognitive enhancers in experimental amnesia. In fact, systemic injection of GRP has been shown to attenuate memory deficits in the scopolamine- and hypoxia-induced models of memory impairment in mice.^40^ We thus decided to examine the effects of GRPR activation by BLPs in an experimental model of memory disorders associated with AD. Rats were given an infusion of a low dose of the neurotoxic fragment of beta-amyloid peptide (Abeta 25-35) into the CA1 hippocampal area. Intrahippocampal administration of Abeta (25-35) produced an impairment of retention of memory for inhibitory avoidance conditioning. GRPR activation by administration of BB to the hippocampus before avoidance training prevented the Abeta (25-35)-induced memory impairment.^16^ This finding indicates that BB and other GRPR agonists might prevent cognitive deficits associated with AD. Table 3 summarizes findings from animal studies supporting the view that drugs acting on the GRPR might display cognitive-enhancing properties.

**Table 3:** Findings from preclinical studies indicating that drugs acting at the gastrin-releasing peptide receptor (GRPR) can display cognitive-enhancing properties.

Species	Finding	References
Rat	Memory enhancement by systemic administration of bombesin (BB) or gastrin-releasing peptide (GRP)	^[26, 27]^
Rat	Enhancement of fear memory by intrahippocampal infusion of BB	^[16]^
Rat	Memory enhancement by infusion of BB into the nucleus tractus solitarius (NTS)	^[38]^
Rat	Enhancement of fear memory by intrahippocampal infusion of an administration of a GRP receptor (GRPR) antagonist	^[33]^
Rat	Enhancement of fear memory by intraamygdala infusion of a GRPR antagonist	^[35]^
Mouse	Enhancement of fear memory and synaptic plasticity in GRPR-deficient knockout mice	^[39]^
Mouse	Improvement of scopolamine and hypoxia-induced amnesia by systemic administration of GRP	^[40]^
Rat	Prevention of memory impairment induced by beta-amyloid peptide (25-35) by intrahippocampal infu-sion of BB	^[16]^

## Perspectives on the clinical use of drugs acting at the GRPR as cognitive enhancers in patients with Alzheimer’s disease

The data reviewed above can be summarized as follows:

(1) the human BLP, GRP, and its receptor, GRPR, are expressed in neurons, and particularly high densities of GRP and GRPR occur in brain areas importantly involved in cognitive function and dementia, such as the hippocampus;
(2) evidence from mouse and human studies suggest that abnormalities in GRPR expression and aspects of GRPR signaling relevant for neurodegeneration and cognitive function (i.e., cellular calcium homeostasis, oxidative stress) might be associated with AD;
(3) preclinical studies show that GRP and the GRPR are importantly involved in regulating synaptic plasticity and memory formation in the hippocampus and other brain areas; and
(4) GRPR agonists can prevent memory disorders in a rat model of amnesia associated with AD.

Together, these findings constitute a consistent body of evidence supporting the view that drugs acting at the GRPR should be further evaluated as potential cognitive enhancers to treat memory disorders associated with AD and other neurodegenerative and psychiatric disorders. In addition to the amphibian and mammalian BLPs that act as GRPR agonists, namely BB and GRP, we have recently shown that the BB analog and GRPR antagonist RC-3095 can also enhance memory when given at high doses to rats.^33^ Thus, both naturally-occurring BLPs and synthetic BB analogs, might display cognitive-enhancing properties and could be considered candidate drugs for the treatment of memory disorders. In addition, our recent findings that the GRPR modulates inflammatory responses,^41^ raises the possibility that GRPR ligands could display neuroprotective actions in addition to facilitating memory in AD patients. Since previous clinical studies in the fields of gastroenterology and oncology have indicated that BLPs and RC-3095 do not induce overt side effects when administered intravenously in humans,^42,43^ clinical trials evaluating the effects of drugs acting at the GRPR on cognitive function in patients with AD and other neurodegenerative and psychiatric disorders are warranted.
